# Bis(diphenyl­phospho­rothio­yl) tris­ulfide

**DOI:** 10.1107/S1600536808024070

**Published:** 2008-08-06

**Authors:** Monika Kulcsar, Anca Silvestru, Marius Vonas

**Affiliations:** a Faculty of Chemistry and Chemical Engineering, Babes-Bolyai University, Arany Janos Street 11, RO-400028 Cluj-Napoca, Romania

## Abstract

In the title compound, C_24_H_20_P_2_S_5_, the P atoms are arranged *trans* with respect to the S_3_ group and the S=P—S—S systems have *cisoid* geometry, with an average S—P—S—S torsion angle of −56.7°. The dihedral angles between the two phenyl rings attached to the P atoms are 87.33 (12) and 75.67 (10)°. In the crystal structure, the mol­ecules are linked into chains running parallel to the *a* axis by weak inter­molecular C—H⋯S hydrogen bonds. Centrosymmetrically related chains are further connected by π–π stacking inter­actions, with a centroid-to-centroid distance of 3.795 (5) Å.

## Related literature

For related literature. see: Deleanu *et al.* (2002 ▶); Drake *et al.* (2001*a* ▶,*b* ▶); Gallacher & Pinkerton (1992*a* ▶,*b* ▶, 1993 ▶); Kulcsar *et al.* (2005 ▶); Newton *et al.* (1993 ▶); Silvestru *et al.* (1994*a* ▶,*b* ▶); Buranda *et al.* (1991 ▶); Fest & Schmidt (1982 ▶); Knopik *et al.* (1993 ▶); Lawton (1970 ▶); McCleverty *et al.* (1983 ▶); Molyneux (1967 ▶); Perlikowska *et al.* (2004 ▶); Potrzebowski *et al.* (1991 ▶, 1994 ▶); Tiekink (2001 ▶); Tkachev *et al.* (1976 ▶); Zhang *et al.* (2004 ▶); Emsley (1994 ▶); Yadav *et al.* (1989 ▶).
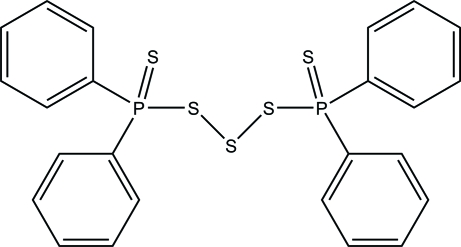


## Experimental

### 

#### Crystal data

C_24_H_20_P_2_S_5_
*M* *_r_* = 530.64
Triclinic, 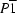

*a* = 9.2287 (8) Å
*b* = 11.5476 (10) Å
*c* = 12.9728 (12) Å
α = 92.690 (2)°
β = 105.287 (2)°
γ = 106.124 (2)°
*V* = 1270.3 (2) Å^3^
*Z* = 2
Mo *K*α radiation
μ = 0.59 mm^−1^
*T* = 297 (2) K
0.35 × 0.27 × 0.21 mm

#### Data collection

Bruker SMART APEX diffractometer
Absorption correction: multi-scan (*SADABS*; Bruker, 2000 ▶) *T*_min_ = 0.819, *T*_max_ = 0.885
13712 measured reflections
5178 independent reflections
4536 reflections with *I* > 2σ(*I*)
*R*_int_ = 0.029

#### Refinement

*R*[*F* ^2^ > 2σ(*F* ^2^)] = 0.047
*wR*(*F* ^2^) = 0.106
*S* = 1.13
5178 reflections
280 parameters
H-atom parameters constrained
Δρ_max_ = 0.40 e Å^−3^
Δρ_min_ = −0.19 e Å^−3^

### 

Data collection: *SMART* (Bruker, 2000 ▶); cell refinement: *SAINT-Plus* (Bruker, 2001 ▶); data reduction: *SAINT-Plus*; program(s) used to solve structure: *SHELXS97* (Sheldrick, 2008 ▶); program(s) used to refine structure: *SHELXL97* (Sheldrick, 2008 ▶); molecular graphics: *DIAMOND* (Brandenburg & Putz, 2006 ▶); software used to prepare material for publication: *publCIF* (Westrip, 2008 ▶).

## Supplementary Material

Crystal structure: contains datablocks I, global. DOI: 10.1107/S1600536808024070/rz2237sup1.cif

Structure factors: contains datablocks I. DOI: 10.1107/S1600536808024070/rz2237Isup2.hkl

Additional supplementary materials:  crystallographic information; 3D view; checkCIF report

## Figures and Tables

**Table 1:** Hydrogen-bond geometry (Å, °)

*D*—H⋯*A*	*D*—H	H⋯*A*	*D*⋯*A*	*D*—H⋯*A*
C17—H17⋯S5^i^	0.93	2.94	3.737 (3)	145

Symmetry code: (i) 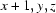.

**Fig. 1.**
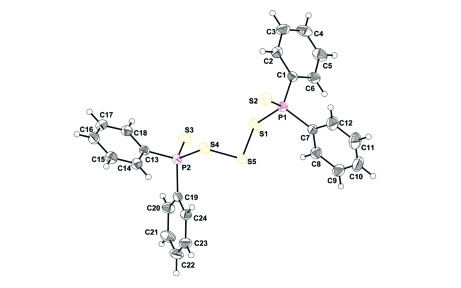
A view of the title compound, with the atomic numbering scheme, showing displacement ellipsoids at the 50% probability level. H atoms are drawn as spheres of arbitrary radii.

**Fig. 2.**
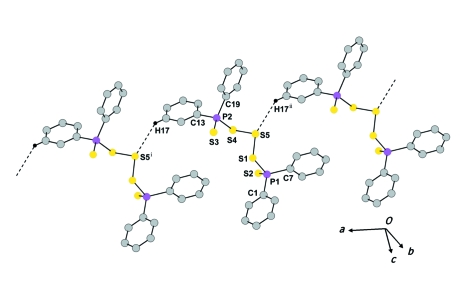
View of the S···H intermolecular interactions (dashed lines) in the title compound. Only H involved in hydrogen bonding interactions are shown. Symmetry codes: (i) 1 + x, y, z, (ii) -1 + x, y, z.

## supplementary crystallographic information

### Comment

Our interest was focused for long time on studies concerning reactions between bis(diorganothiophosphoryl)disulfides and diorganodichalcogenides of type *R*_2_E_2_ (E = Se, Te) in order to obtain new organochalcogen compounds with diorganodithiophosphorus ligands (Newton *et al.*, 1993; Silvestru *et al.*, 1994*a*,*b*; Drake *et al.*, 2001*a*,*b*; Kulcsar *et al.*, 2005; Deleanu *et al.*, 2002). Bis(diorganothiophosphoryl)disulfides attracted much interest also due to their potential applications as pesticides (Fest & Schmidt, 1982), additives in motor oils (Molyneux, 1967) and in the rubber vulcanization (McCleverty *et al.*, 1983). Both alkyl and aryl substituted derivatives of type [*R*_2_P(S)S]_2_ were already structurally characterized, *e.g. R* = OPr-i (Lawton, 1970; Tkachev *et al.*, 1976; Tiekink, 2001; Zhang *et al.*, 2004), *R* = OMe, OBu-t (Potrzebowski *et al.*, 1991), cyclohexyl (Buranda *et al.*, 1991), Me, Pr-i (Gallacher & Pinkerton, 1992*b*), Ph (Gallacher & Pinkerton, 1993), OPh (Gallacher & Pinkerton, 1993; Knopik *et al.*, 1993), menthoxy (Perlikowska *et al.*, 2004), *R*_2_ = OCMe_2_—CMe_2_O (Yadav *et al.*, 1989), OCMe_2_—CH_2_O (Potrzebowski *et al.*, 1994). A search of the Cambridge Structure Database revealed that only for one trisulfide, [Et_2_P(S)S]_2_S, the X-ray crystal structure was determined (Gallacher & Pinkerton, 1992*a*). Here we report about the phenyl substituted analog, [Ph_2_P(S)S]_2_S.

The chalcogen atoms S2 and S3 are doubly bonded to phosphorus [S2═P1 = 1.9351 (9); S3═P2 = 1.9303 (9) Å], while the P1—S1 [2.1171 (9) Å] and P2—S4 [2.1282 (9) Å] distances correspond to single P—S bonds (*cf.* [Ph_2_P(S)S]_2_: P═S = 1.930 (1), P—S = 2.139 (1) Å; Gallacher & Pinkerton, 1993). The sulfur–sulfur distances within the S_3_ group are not significantly different [S5—S4 = 2.0407 (10), S5—S1 = 2.0440 (10) Å], corresponding to a S—S single bond. These values are similar to those found in bis(diorganothiophosphoryl)disulfides or in [Et_2_P(S)S]_2_S. The SPS_3_PS skeleton of the title compound adopts a twisted zigzag chain structure (Fig. 1), with the torsion angles S2—P1—S1—S5 = -56.30 (5)°, P1—S1—S5—S4 = 96.84 (4)°, S1—S5—S4—P2 = 87.42 (5)° and S5—S4—P2—S3 = -57.13 (5)°. Although apparently the conformation of the SPS_3_PS skeleton is similar to that of the previously reported ethyl derivative, some differences should be noted. In the title compound the phosphorus atoms are *trans* with respect of the central S_3_ group [P1—S1···S5—P2 = 171.4°], as are in the related [Et_2_P(S)S]_2_S compound (the torsion angle between corresponding atoms is 159.8°). In both cases the central S atom and the terminal S atoms, respectively, are placed on opposite sides of the best plane described by the remaining atoms of the skeleton. However, the S═P···P═S torsion angle is 138.8°, but only -89.4° in the ethyl derivative. Moreover, the S═P—S—S system has a *cisoid* geometry [S2—P1—S1—S5 = -56.30 (5)°, S5—S4—P2—S3 = -57.13 (5)°], while it has a *transoid* geometry in [Et_2_P(S)S]_2_S (average S═P—S—S torsion angle 179.6°; Gallacher & Pinkerton, 1992*a*). The S—P—S angles [S2—P1—S1 = 113.77 (4)° and S3—P2—S4 = 114.34 (4)°] are consistent with a *cisoid* geometry, similar with that found for [Ph_2_P(S)S]_2_ [114.44 (4)°; Gallacher & Pinkerton, 1993], but much larger than in the *transoid* derivatives [(PhO)_2_P(S)S]_2_ [108.39 (7)°; Gallacher & Pinkerton, 1993] and [Et_2_P(S)S]_2_S [av. 103.7°; Gallacher & Pinkerton, 1992*a*]. The dihedral angles formed by the plane of the phenyl rings attached to the P1 and P2 atoms are 87.33 (12) 75.67 (10)° respectively. The crystal structure is stabilized by weak intermolecular hydrogen bonding interactions (Emsley, 1994) between the central sulfur atom and an aromatic proton of a neighbouring molecule (Table 1) forming chains parellel to the *a* axis (Fig. 2). Weak intermolecular S···H contacts were observed in [(PhO)_2_P(S)S]_2_ [2.954 (1) Å], but they are absent in [Et_2_P(S)S]_2_S or [Ph_2_P(S)S]_2_. Centrosymmetrically related chains are further connected by π–π stacking interactions involving the C13–C18 phenyl rings, with centroid-to-centroid distance of 3.795 (5) Å.

### Experimental

The title compound was isolated as a by-product during recrystallization of PhSeS_2_PPh_2_ obtained in the reaction between [Ph_2_P(S)S]_2_ and *Ph*_2_Se_2_.

### Refinement

All C-bound H atoms were placed in calculated positions (C—H = 0.93–0.97 Å) and treated using a riding model with *U*_iso_ = 1.2*U*_eq_(C).

### Crystal data

**Table:**

C_24_H_20_P_2_S_5_	*Z* = 2
*M**_r_* = 530.64	*F*_000_ = 548
Triclinic, *P*1	*D*_x_ = 1.387 Mg m^−^^3^
Hall symbol: -P 1	Mo *K*α radiation λ = 0.71073 Å
*a* = 9.2287 (8) Å	Cell parameters from 4229 reflections
*b* = 11.5476 (10) Å	θ = 2.4–25.3º
*c* = 12.9728 (12) Å	µ = 0.59 mm^−^^1^
α = 92.690 (2)º	*T* = 297 (2) K
β = 105.287 (2)º	Block, yellow
γ = 106.124 (2)º	0.35 × 0.27 × 0.21 mm
*V* = 1270.3 (2) Å^3^	

### Data collection

**Table:**

Bruker SMART APEX diffractometer	5178 independent reflections
Radiation source: fine-focus sealed tube	4536 reflections with *I* > 2σ(*I*)
Monochromator: graphite	*R*_int_ = 0.029
*T* = 297(2) K	θ_max_ = 26.4º
φ and ω scans	θ_min_ = 1.6º
Absorption correction: multi-scan(SADABS; Bruker, 2000)	*h* = −11→11
*T*_min_ = 0.819, *T*_max_ = 0.886	*k* = −14→14
13712 measured reflections	*l* = −16→16

### Refinement

**Table:**

Refinement on *F*^2^	Secondary atom site location: difference Fourier map
Least-squares matrix: full	Hydrogen site location: inferred from neighbouring sites
*R*[*F*^2^ > 2σ(*F*^2^)] = 0.047	H-atom parameters constrained
*wR*(*F*^2^) = 0.106	*w* = 1/[σ^2^(*F*_o_^2^) + (0.0406*P*)^2^ + 0.4205*P*] where *P* = (*F*_o_^2^ + 2*F*_c_^2^)/3
*S* = 1.13	(Δ/σ)_max_ = 0.001
5178 reflections	Δρ_max_ = 0.40 e Å^−^^3^
280 parameters	Δρ_min_ = −0.19 e Å^−^^3^
Primary atom site location: structure-invariant direct methods	Extinction correction: none

### Special details

**Table:**

Geometry. All e.s.d.'s (except the e.s.d. in the dihedral angle between two l.s. planes) are estimated using the full covariance matrix. The cell e.s.d.'s are taken into account individually in the estimation of e.s.d.'s in distances, angles and torsion angles; correlations between e.s.d.'s in cell parameters are only used when they are defined by crystal symmetry. An approximate (isotropic) treatment of cell e.s.d.'s is used for estimating e.s.d.'s involving l.s. planes.
Refinement. Refinement of *F*^2^ against ALL reflections. The weighted *R*-factor *wR* and goodness of fit *S* are based on *F*^2^, conventional *R*-factors *R* are based on *F*, with *F* set to zero for negative *F*^2^. The threshold expression of *F*^2^ > σ(*F*^2^) is used only for calculating *R*-factors(gt) *etc*. and is not relevant to the choice of reflections for refinement. *R*-factors based on *F*^2^ are statistically about twice as large as those based on *F*, and *R*- factors based on ALL data will be even larger.

### Fractional atomic coordinates and isotropic or equivalent isotropic displacement parameters (Å^2^)

**Table:**

	*x*	*y*	*z*	*U*_iso_*/*U*_eq_	
C1	0.2158 (3)	0.8016 (2)	1.01664 (19)	0.0410 (6)	
C2	0.3749 (3)	0.8562 (3)	1.0317 (2)	0.0549 (7)	
H2	0.4141	0.8641	0.9724	0.066*	
C3	0.4758 (4)	0.8988 (3)	1.1337 (3)	0.0649 (8)	
H3	0.5828	0.9348	1.1436	0.078*	
C4	0.4171 (4)	0.8878 (3)	1.2203 (3)	0.0689 (9)	
H4	0.4848	0.9168	1.2894	0.083*	
C5	0.2607 (4)	0.8349 (3)	1.2068 (2)	0.0710 (9)	
H5	0.2225	0.8283	1.2665	0.085*	
C6	0.1586 (3)	0.7909 (3)	1.1050 (2)	0.0561 (7)	
H6	0.0520	0.7543	1.0960	0.067*	
C7	−0.1095 (3)	0.7270 (2)	0.8809 (2)	0.0432 (6)	
C8	−0.2183 (3)	0.6199 (3)	0.8876 (2)	0.0541 (7)	
H8	−0.1877	0.5500	0.8980	0.065*	
C9	−0.3720 (4)	0.6158 (4)	0.8789 (3)	0.0721 (10)	
H9	−0.4449	0.5435	0.8832	0.087*	
C10	−0.4163 (4)	0.7184 (5)	0.8640 (3)	0.0890 (12)	
H10	−0.5203	0.7156	0.8568	0.107*	
C11	−0.3101 (5)	0.8242 (4)	0.8595 (4)	0.0986 (13)	
H11	−0.3412	0.8941	0.8513	0.118*	
C12	−0.1564 (4)	0.8300 (3)	0.8671 (3)	0.0735 (9)	
H12	−0.0848	0.9030	0.8629	0.088*	
C13	0.3127 (3)	0.3345 (2)	0.5892 (2)	0.0398 (5)	
C14	0.2557 (3)	0.3370 (2)	0.4795 (2)	0.0479 (6)	
H14	0.1519	0.3359	0.4499	0.057*	
C15	0.3525 (4)	0.3410 (3)	0.4141 (2)	0.0556 (7)	
H15	0.3143	0.3436	0.3406	0.067*	
C16	0.5055 (4)	0.3413 (3)	0.4577 (3)	0.0609 (8)	
H16	0.5705	0.3430	0.4135	0.073*	
C17	0.5620 (3)	0.3390 (3)	0.5657 (3)	0.0619 (8)	
H17	0.6657	0.3395	0.5946	0.074*	
C18	0.4675 (3)	0.3360 (2)	0.6329 (2)	0.0514 (7)	
H18	0.5072	0.3349	0.7065	0.062*	
C19	0.0085 (3)	0.2000 (2)	0.61159 (19)	0.0391 (5)	
C20	0.0029 (3)	0.0881 (2)	0.6477 (2)	0.0553 (7)	
H20	0.0863	0.0805	0.7028	0.066*	
C21	−0.1260 (4)	−0.0116 (3)	0.6021 (3)	0.0679 (9)	
H21	−0.1291	−0.0866	0.6261	0.082*	
C22	−0.2495 (4)	−0.0006 (3)	0.5214 (3)	0.0663 (9)	
H22	−0.3370	−0.0679	0.4914	0.080*	
C23	−0.2445 (3)	0.1094 (3)	0.4849 (2)	0.0590 (8)	
H23	−0.3284	0.1163	0.4297	0.071*	
C24	−0.1155 (3)	0.2102 (2)	0.5295 (2)	0.0468 (6)	
H24	−0.1123	0.2846	0.5042	0.056*	
P1	0.09047 (8)	0.73820 (6)	0.88233 (5)	0.03962 (16)	
P2	0.18502 (8)	0.32528 (6)	0.67495 (5)	0.03861 (16)	
S1	0.11328 (9)	0.56101 (6)	0.88821 (5)	0.04965 (18)	
S2	0.15246 (9)	0.81659 (7)	0.76545 (6)	0.0581 (2)	
S3	0.27532 (9)	0.31611 (7)	0.82540 (5)	0.0562 (2)	
S4	0.12601 (8)	0.48837 (6)	0.64407 (5)	0.04720 (18)	
S5	−0.02304 (8)	0.48041 (6)	0.73690 (6)	0.04856 (18)	

### Atomic displacement parameters (Å^2^)

**Table:**

	*U*^11^	*U*^22^	*U*^33^	*U*^12^	*U*^13^	*U*^23^
C1	0.0431 (14)	0.0406 (13)	0.0377 (13)	0.0128 (11)	0.0096 (11)	0.0023 (10)
C2	0.0462 (15)	0.0608 (18)	0.0556 (17)	0.0146 (13)	0.0134 (13)	0.0027 (14)
C3	0.0443 (16)	0.064 (2)	0.072 (2)	0.0126 (14)	−0.0026 (15)	0.0023 (16)
C4	0.076 (2)	0.0575 (19)	0.0514 (18)	0.0143 (17)	−0.0112 (16)	−0.0003 (14)
C5	0.089 (3)	0.074 (2)	0.0389 (16)	0.0113 (19)	0.0159 (16)	0.0006 (15)
C6	0.0559 (17)	0.0611 (18)	0.0435 (15)	0.0060 (14)	0.0147 (13)	0.0017 (13)
C7	0.0407 (13)	0.0489 (15)	0.0380 (13)	0.0129 (11)	0.0096 (11)	−0.0008 (11)
C8	0.0533 (16)	0.0624 (18)	0.0486 (16)	0.0170 (14)	0.0183 (13)	0.0084 (13)
C9	0.0529 (18)	0.094 (3)	0.061 (2)	0.0040 (18)	0.0249 (16)	−0.0066 (18)
C10	0.055 (2)	0.131 (4)	0.088 (3)	0.040 (2)	0.0229 (19)	−0.009 (3)
C11	0.085 (3)	0.093 (3)	0.137 (4)	0.056 (3)	0.034 (3)	0.007 (3)
C12	0.067 (2)	0.0587 (19)	0.103 (3)	0.0281 (17)	0.0295 (19)	0.0056 (18)
C13	0.0425 (13)	0.0311 (12)	0.0428 (14)	0.0077 (10)	0.0113 (11)	0.0010 (10)
C14	0.0475 (15)	0.0504 (15)	0.0453 (15)	0.0136 (12)	0.0138 (12)	0.0076 (12)
C15	0.0659 (19)	0.0535 (17)	0.0465 (16)	0.0101 (14)	0.0232 (14)	0.0062 (13)
C16	0.0594 (19)	0.0526 (17)	0.072 (2)	0.0048 (14)	0.0353 (17)	−0.0010 (15)
C17	0.0399 (15)	0.0612 (19)	0.078 (2)	0.0090 (13)	0.0144 (15)	−0.0046 (16)
C18	0.0474 (15)	0.0493 (16)	0.0510 (16)	0.0111 (13)	0.0081 (13)	−0.0013 (12)
C19	0.0442 (13)	0.0345 (12)	0.0406 (13)	0.0104 (10)	0.0173 (11)	0.0027 (10)
C20	0.0587 (17)	0.0399 (15)	0.0614 (18)	0.0117 (13)	0.0107 (14)	0.0075 (13)
C21	0.083 (2)	0.0376 (16)	0.072 (2)	0.0061 (15)	0.0157 (18)	0.0063 (14)
C22	0.068 (2)	0.0485 (17)	0.064 (2)	−0.0070 (15)	0.0186 (17)	−0.0096 (15)
C23	0.0486 (16)	0.0630 (19)	0.0515 (17)	0.0052 (14)	0.0052 (13)	−0.0032 (14)
C24	0.0480 (15)	0.0451 (15)	0.0454 (15)	0.0117 (12)	0.0129 (12)	0.0061 (12)
P1	0.0411 (4)	0.0412 (4)	0.0361 (3)	0.0117 (3)	0.0113 (3)	0.0044 (3)
P2	0.0440 (4)	0.0349 (3)	0.0359 (3)	0.0113 (3)	0.0105 (3)	0.0039 (3)
S1	0.0608 (4)	0.0496 (4)	0.0432 (4)	0.0259 (3)	0.0128 (3)	0.0062 (3)
S2	0.0642 (5)	0.0640 (5)	0.0451 (4)	0.0116 (4)	0.0206 (3)	0.0161 (3)
S3	0.0658 (5)	0.0598 (4)	0.0378 (4)	0.0173 (4)	0.0073 (3)	0.0082 (3)
S4	0.0632 (4)	0.0346 (3)	0.0467 (4)	0.0161 (3)	0.0191 (3)	0.0064 (3)
S5	0.0403 (3)	0.0436 (4)	0.0562 (4)	0.0096 (3)	0.0097 (3)	−0.0040 (3)

### Geometric parameters (Å, °)

**Table:**

C1—C6	1.380 (4)	C14—C15	1.378 (4)
C1—C2	1.384 (4)	C14—H14	0.9300
C1—P1	1.805 (2)	C15—C16	1.375 (4)
C2—C3	1.377 (4)	C15—H15	0.9300
C2—H2	0.9300	C16—C17	1.365 (4)
C3—C4	1.367 (5)	C16—H16	0.9300
C3—H3	0.9300	C17—C18	1.382 (4)
C4—C5	1.361 (5)	C17—H17	0.9300
C4—H4	0.9300	C18—H18	0.9300
C5—C6	1.380 (4)	C19—C24	1.379 (3)
C5—H5	0.9300	C19—C20	1.387 (3)
C6—H6	0.9300	C19—P2	1.816 (2)
C7—C12	1.379 (4)	C20—C21	1.376 (4)
C7—C8	1.383 (4)	C20—H20	0.9300
C7—P1	1.809 (3)	C21—C22	1.369 (4)
C8—C9	1.381 (4)	C21—H21	0.9300
C8—H8	0.9300	C22—C23	1.369 (4)
C9—C10	1.362 (5)	C22—H22	0.9300
C9—H9	0.9300	C23—C24	1.383 (4)
C10—C11	1.353 (6)	C23—H23	0.9300
C10—H10	0.9300	C24—H24	0.9300
C11—C12	1.378 (5)	P1—S2	1.9351 (9)
C11—H11	0.9300	P1—S1	2.1171 (9)
C12—H12	0.9300	P2—S3	1.9303 (9)
C13—C14	1.385 (3)	P2—S4	2.1282 (9)
C13—C18	1.386 (4)	S1—S5	2.0440 (10)
C13—P2	1.808 (2)	S4—S5	2.0407 (10)
			
C6—C1—C2	119.4 (2)	C14—C15—H15	120.1
C6—C1—P1	121.7 (2)	C17—C16—C15	120.0 (3)
C2—C1—P1	118.9 (2)	C17—C16—H16	120.0
C3—C2—C1	120.5 (3)	C15—C16—H16	120.0
C3—C2—H2	119.8	C16—C17—C18	121.0 (3)
C1—C2—H2	119.8	C16—C17—H17	119.5
C4—C3—C2	119.4 (3)	C18—C17—H17	119.5
C4—C3—H3	120.3	C17—C18—C13	119.1 (3)
C2—C3—H3	120.3	C17—C18—H18	120.4
C5—C4—C3	120.8 (3)	C13—C18—H18	120.4
C5—C4—H4	119.6	C24—C19—C20	119.5 (2)
C3—C4—H4	119.6	C24—C19—P2	123.69 (19)
C4—C5—C6	120.4 (3)	C20—C19—P2	116.8 (2)
C4—C5—H5	119.8	C21—C20—C19	120.1 (3)
C6—C5—H5	119.8	C21—C20—H20	120.0
C1—C6—C5	119.6 (3)	C19—C20—H20	120.0
C1—C6—H6	120.2	C22—C21—C20	120.2 (3)
C5—C6—H6	120.2	C22—C21—H21	119.9
C12—C7—C8	118.9 (3)	C20—C21—H21	119.9
C12—C7—P1	117.5 (2)	C23—C22—C21	120.1 (3)
C8—C7—P1	123.5 (2)	C23—C22—H22	119.9
C9—C8—C7	120.5 (3)	C21—C22—H22	119.9
C9—C8—H8	119.8	C22—C23—C24	120.4 (3)
C7—C8—H8	119.8	C22—C23—H23	119.8
C10—C9—C8	119.6 (3)	C24—C23—H23	119.8
C10—C9—H9	120.2	C19—C24—C23	119.8 (3)
C8—C9—H9	120.2	C19—C24—H24	120.1
C11—C10—C9	120.4 (3)	C23—C24—H24	120.1
C11—C10—H10	119.8	C1—P1—C7	107.49 (11)
C9—C10—H10	119.8	C1—P1—S2	116.30 (9)
C10—C11—C12	120.9 (4)	C7—P1—S2	113.64 (9)
C10—C11—H11	119.5	C1—P1—S1	96.94 (8)
C12—C11—H11	119.5	C7—P1—S1	107.14 (9)
C11—C12—C7	119.6 (3)	S2—P1—S1	113.77 (4)
C11—C12—H12	120.2	C13—P2—C19	106.48 (11)
C7—C12—H12	120.2	C13—P2—S3	116.70 (9)
C14—C13—C18	119.7 (2)	C19—P2—S3	113.09 (8)
C14—C13—P2	120.46 (19)	C13—P2—S4	97.91 (8)
C18—C13—P2	119.8 (2)	C19—P2—S4	106.85 (8)
C15—C14—C13	120.2 (3)	S3—P2—S4	114.34 (4)
C15—C14—H14	119.9	S5—S1—P1	100.37 (4)
C13—C14—H14	119.9	S5—S4—P2	99.52 (4)
C16—C15—C14	119.9 (3)	S4—S5—S1	106.77 (4)
C16—C15—H15	120.1		
			
C6—C1—C2—C3	0.4 (4)	C6—C1—P1—C7	24.6 (3)
P1—C1—C2—C3	−176.0 (2)	C2—C1—P1—C7	−159.0 (2)
C1—C2—C3—C4	−0.6 (5)	C6—C1—P1—S2	153.3 (2)
C2—C3—C4—C5	0.3 (5)	C2—C1—P1—S2	−30.4 (2)
C3—C4—C5—C6	0.3 (5)	C6—C1—P1—S1	−85.9 (2)
C2—C1—C6—C5	0.1 (4)	C2—C1—P1—S1	90.5 (2)
P1—C1—C6—C5	176.4 (2)	C12—C7—P1—C1	82.6 (2)
C4—C5—C6—C1	−0.4 (5)	C8—C7—P1—C1	−100.7 (2)
C12—C7—C8—C9	1.0 (4)	C12—C7—P1—S2	−47.6 (3)
P1—C7—C8—C9	−175.7 (2)	C8—C7—P1—S2	129.2 (2)
C7—C8—C9—C10	−0.2 (5)	C12—C7—P1—S1	−174.1 (2)
C8—C9—C10—C11	−1.2 (6)	C8—C7—P1—S1	2.6 (2)
C9—C10—C11—C12	1.7 (7)	C14—C13—P2—C19	50.3 (2)
C10—C11—C12—C7	−0.9 (6)	C18—C13—P2—C19	−128.0 (2)
C8—C7—C12—C11	−0.4 (5)	C14—C13—P2—S3	177.66 (17)
P1—C7—C12—C11	176.5 (3)	C18—C13—P2—S3	−0.6 (2)
C18—C13—C14—C15	−0.1 (4)	C14—C13—P2—S4	−59.9 (2)
P2—C13—C14—C15	−178.4 (2)	C18—C13—P2—S4	121.75 (19)
C13—C14—C15—C16	0.7 (4)	C24—C19—P2—C13	−82.9 (2)
C14—C15—C16—C17	−0.8 (4)	C20—C19—P2—C13	95.5 (2)
C15—C16—C17—C18	0.2 (5)	C24—C19—P2—S3	147.60 (19)
C16—C17—C18—C13	0.4 (4)	C20—C19—P2—S3	−34.0 (2)
C14—C13—C18—C17	−0.4 (4)	C24—C19—P2—S4	20.9 (2)
P2—C13—C18—C17	177.9 (2)	C20—C19—P2—S4	−160.63 (19)
C24—C19—C20—C21	−0.3 (4)	C1—P1—S1—S5	−179.08 (9)
P2—C19—C20—C21	−178.8 (2)	C7—P1—S1—S5	70.17 (9)
C19—C20—C21—C22	−0.4 (5)	S2—P1—S1—S5	−56.30 (5)
C20—C21—C22—C23	0.8 (5)	C13—P2—S4—S5	178.76 (8)
C21—C22—C23—C24	−0.5 (5)	C19—P2—S4—S5	68.80 (9)
C20—C19—C24—C23	0.7 (4)	S3—P2—S4—S5	−57.13 (5)
P2—C19—C24—C23	179.1 (2)	P2—S4—S5—S1	87.43 (4)
C22—C23—C24—C19	−0.3 (4)	P1—S1—S5—S4	96.84 (4)

### Hydrogen-bond geometry (Å, °)

**Table:**

*D*—H···*A*	*D*—H	H···*A*	*D*···*A*	*D*—H···*A*
C17—H17···S5^i^	0.93	2.94	3.737 (3)	145

Symmetry codes: (i) *x*+1, *y*, *z*.

## References

[bb1] Brandenburg, K. & Putz, H. (2006). *DIAMOND* Crystal Impact GbR, Bonn, Germany.

[bb2] Bruker (2000). *SMART* and *SADABS* Bruker AXS Inc., Madison, Wisconsin, USA.

[bb3] Bruker (2001). *SAINT-Plus* Bruker AXS Inc., Madison, Wisconsin, USA.

[bb4] Buranda, T., Gallacher, A. C. & Pinkerton, A. A. (1991). *Acta Cryst.* C**47**, 1414–1418.

[bb5] Deleanu, C., Drake, J. E., Hursthouse, M. B., Kulcsar, M., Leight, M. E. & Silvestru, A. (2002). *Appl. Organomet. Chem.* **16**, 727–731.

[bb6] Drake, J. E., Hursthouse, M. B., Kulcsar, M., Light, M. E. & Silvestru, A. (2001*a*). *Phosphorus Sulfur Silicon Relat. Elem.* **169**, 293–296.

[bb7] Drake, J. E., Hursthouse, M. B., Kulcsar, M., Light, M. E. & Silvestru, A. (2001*b*). *J. Organomet. Chem.* **623**, 153–160.

[bb8] Emsley, J. (1994). *Die Elemente* Berlin: Walter de Gruyter.

[bb9] Fest, C. & Schmidt, K. J. (1982). *The Chemistry of Organophosphorus Pesticides*, 2nd ed. New York: Springer.

[bb10] Gallacher, A. C. & Pinkerton, A. A. (1992*a*). *Acta Cryst.* C**48**, 701–703.

[bb11] Gallacher, A. C. & Pinkerton, A. A. (1992*b*). *Acta Cryst.* C**48**, 2085–2088.

[bb12] Gallacher, A. C. & Pinkerton, A. A. (1993). *Acta Cryst.* C**49**, 1793–1796.

[bb13] Knopik, P., Luczak, L., Potrzebowski, M. J., Michalski, J., Blaszczyk, J. & Wieczorek, M. W. (1993). *J. Chem. Soc. Dalton Trans.* pp. 2749–2757.

[bb14] Kulcsar, M., Silvestru, A., Silvestru, C., Drake, J. E., Macdonald, C. L. B., Hursthouse, M. B. & Light, M. E. (2005). *J. Organomet. Chem.* **690**, 3217–3228.

[bb15] Lawton, S. L. (1970). *Inorg. Chem.* **9**, 2269–2274.

[bb16] McCleverty, J. A., Kowalski, R. S. Z., Bailey, N. A., Mulvaney, R. & O’Cleirigh, D. A. (1983). *J. Chem. Soc. Dalton Trans.* pp. 627–634.

[bb17] Molyneux, P. H. (1967). *Lubrication and Lubricants*, ch. 3, edited by E. R. Braithwaite. Amsterdam: Elsevier.

[bb18] Newton, M. G., King, R. B., Haiduc, I. & Silvestru, A. (1993). *Inorg. Chem.* **32**, 3795–3796.

[bb19] Perlikowska, W., Gouygou, M., Mikolajczyk, M. & Daran, J.-C. (2004). *Tetrahedron Asymmetry*, **15**, 3519–3529.

[bb20] Potrzebowski, M. J., Grossmann, G., Blaszczyk, J., Wieczorek, M. W., Sieler, J., Knopik, P. & Komber, H. (1994). *Inorg. Chem.* **33**, 4688–4695.

[bb21] Potrzebowski, M. J., Reinbenspies, J. H. & Zhong, Z. (1991). *Heteroat. Chem.* **2**, 455–460.

[bb22] Sheldrick, G. M. (2008). *Acta Cryst.* A**64**, 112–122. 10.1107/S0108767307043930 18156677

[bb23] Silvestru, A., Haiduc, I., Ebert, K. H. & Breunig, H. J. (1994*a*). *Inorg. Chem.* **33**, 1253–1254.

[bb24] Silvestru, A., Haiduc, I., Ebert, K. H. & Breunig, H. J. (1994*b*). *J. Organomet. Chem.* **482**, 253–259.

[bb25] Tiekink, E. R. T. (2001). *Z. Kristallogr. New Cryst. Struct.* **216**, 247–248.

[bb26] Tkachev, V. V., Atovmyan, L. O. & Shchepinov, S. A. (1976). *Zh. Strukt. Khim.* **17**, 945–947.

[bb27] Westrip, S. P. (2008). *publCIF* In preparation.

[bb28] Yadav, J. S., Bohra, R., Mehrotra, R. K., Rai, A. K. & Srivastava, G. (1989). *Acta Cryst.* C**45**, 308–311.

[bb29] Zhang, S.-S., Li, X.-M., Wang, J.-L., Wan, J. & Jiao, K. (2004). *Chem. Res. Chin. Univ.* **20**, 146–148.

